# 3D Printing With Low-Cost and Recycled Medical-Grade Plastics: A Narrative Review of a Sustainable Pathway for Neurosurgical Tools, Training Models, and Implants in Low- and Middle-Income Countries

**DOI:** 10.7759/cureus.114822

**Published:** 2026-08-19

**Authors:** Zia-Ul-Rehman Najeeb, Muhammad Fawad Ul Hassan, Faiqa I Khan, Muhammad Farhan, Waleed Usman, Hunzla A Wajid, Shoaib Ahmad, Ahmad Noor, Sundas Irshad, Haseeb Mehmood Qadri

**Affiliations:** 1 Neurological Surgery, Punjab Institute of Neurosciences, Lahore, PAK; 2 Neurological Surgery, National Hospital and Medical Centre, Lahore, PAK; 3 Neurological Surgery, Aziz Bhatti Shaheed Teaching Hospital, Gujrat, PAK; 4 Internal Medicine, Allama Iqbal Medical College, Lahore, PAK; 5 Neurological Surgery, Shaikh Zayed Medical Complex, Lahore, PAK; 6 Internal Medicine, Shaikh Zayed Hospital, Rahim Yar Khan, PAK; 7 General Surgery, Lahore General Hospital, Lahore, PAK

**Keywords:** 3d modeling, 3d printing, biodegradable, biomaterials, cranioplasty, facial plastic, lmic, low- and middle-income country, neurosurgery, polymer

## Abstract

3D printing enables rapid prototyping and the customized production of functional medical devices. In neurosurgery, where precise anatomical representation is essential, 3D printing is widely utilized to fabricate patient-specific implants, surgical guides, and anatomical models for preoperative planning and training purposes. Medical 3D printing predominantly utilizes polymer-based materials. The advances in polymer science have established biomaterials as essential components in healthcare applications. Healthcare systems in low- and middle-income countries (LMICs) are burdened by financial limitations, rendering the acquisition of expensive, imported medical-grade filaments and biomaterials a significant concern. This narrative review focuses on the use of low-cost and recycled biomaterials and 3D printing in neurosurgery, particularly in LMICs. Additive manufacturing (AM) not only reduces waste concerns but also helps in designing refined products. Keeping the tensile strength, durability, and elastic modulus the same, the integration of low-cost and recycled medical-grade plastics and polymer-based biomaterials offers a practical approach to overcoming these barriers by improving affordability, accessibility, and local manufacturing capacity. 3D printing offers the potential for sustainable innovation in healthcare by utilizing locally available and recycled materials, aligning with cost-effective and environmentally conscious practices.

## Introduction and background

3D printing (“bio-printing”) is an additive manufacturing (AM) method. Besides traditional manufacturing methods such as formative and subtractive, the additive method offers the layer-by-layer successive construction of the product, from a 2D image to a 3D model [1]. It works on the principle of command and action. It receives the commands directly from a computer-aided design (CAD) software [2]. This dynamic innovation is already flourishing in the healthcare market due to its resilience and capability to construct an edifice. In the medical and dental sector, it generated 16.4% of industrial revenue in 2013 [2]. A typical CAD software stores the layout in tessellation format called stereolithography (STL) files. These are the tiniest facets of the whole construct [2]. Other additive models include multi-jet modeling (MJM), fused deposition modeling (FDM), and selective laser melting (SLM) [3].

In fields like neurosurgery, where anatomical accuracy is crucial, 3D printing is being used to produce patient-specific implants, surgical guides, and anatomical models for preoperative planning and surgical training [2]. Biomaterials range from nanoparticles and cell-stable hydrogels to solid metals [4]. In medical 3D printing, polymer-based materials are frequently used, for instance, acrylonitrile butadiene (ABS), polycarbonates (PC), polylactic acid (PLA), polyethene terephthalate glycol (PETG), polyether ether ketone (PEEK), Polyamide 12 (Nylon), and epoxy or methacrylic acid-based materials. The biocompatibility of a product is assessed by the following factors: intended function and the body’s response. Most polymers are also biocompatible, making them safe for use in contact with human tissues. The main point of concern is environmental sustainability. These biomaterials are not readily usable. They go through a process called “extrusion,” through which 3D printing feedstock “filaments” are created [5]. These filaments are expensive, and many manufacturers prefer to extrude filaments from virgin plastic or plastic waste [5]. Current literature lacks a comprehensive assessment (life cycle assessment (LCA)) of the whole AM process, thereby implicating certain environmental concerns. While AM offers many advantages over traditional methods, they are not readily “green.” However, AM offers low energy consumption at the processing level, such as lesser by-product as compared to the subtraction method. Furthermore, some materials are hard to recycle, and the impact of the ultrafine residues is understudied [1-5].

Lower- and middle-income countries (LMICs) face financial difficulties in the healthcare sector, which is why medical-grade filaments and virgin biomaterials are a big concern [2]. When they use implants that are commercially produced, simple procedures such as cranioplasty become very expensive. Therefore, the shift in paradigm to recycling is both durable and convenient. Hospitals also produce a huge amount of medical-grade plastic waste, which is thrown away even if it could be reused. A recent study showed that hospital high-density polyethylene (HDPE) bottle caps can be recycled into 3D printing filament and used to create patient-specific vascular models for neurosurgical planning [5]. It can provide a potentially affordable and environmentally friendly substitute for creating medical tools and models [6].

Recycling medical-grade plastics for 3D printing could offer an innovative solution for healthcare systems. By turning plastic waste into usable printing material, hospitals may be able to produce neurosurgical instruments, educational models, and possibly implants locally at much reduced cost. This narrative review attempts to provide an overview of the feasibility and challenges of using recycled and low-cost biomaterials for 3D printing, especially for lower- and middle-income countries.

## Review

In LMICs, even advanced centers face several challenges. These include cost-effectiveness, safe reusability, and educational training. Firstly, the cost of cranioplasty is highly variable depending on the method and materials used [7]. Titanium and polyetheretherketone (PEEK) implants are preferred in some settings because of their good outcomes but are less frequently used in LMICs due to their high cost [7]. Personalized titanium and PEEK implants can cost up to 10,265 USD and 10,450 USD, respectively, thereby making their accessibility difficult in resource-constrained settings [3]. In contrast, according to a review by Czyżewski et al., a 3D-printed patient-specific polymethylmethacrylate (PMMA) prosthesis costs around 600 USD, which includes designing, prototyping, and final making of an implant, thus reducing overall expenditure by more than 10-fold [3]. However, access to the equipment, materials, software, and post-processing requirements remains very difficult for many medical facilities [8]. Computer-aided design (CAD) or computer-aided manufacturing (CAM) technology produces patient-specific implants but requires industrial-grade printers and specific licenses, creating an additional cost and a barrier for institutions in resource-limited environments [1].

Moreover, insurance agencies and public health administrators usually do not validate custom-made 3D-printed implants, creating a financial burden and affecting patients in lower-income settings [1]. Due to the lack of accessible 3D modeling infrastructure, preoperative planning relies heavily on a conventional workflow, hence limiting the precision and timeliness of reconstructive procedures following decompressive craniectomy [1]. The mean time to cranioplasty among patients in LMIC was 8.5 months, exceeding the recommended threshold of 3 months [7]. An interval of 12-36 months has also been reported in some studies [9]. Prolonged delay in cranioplasty has been associated with increased infection rate and extended antibiotic use, resulting in increased overall costs [10]. This highlights the need for prompt surgical intervention in LMICs [9].

Secondly, the contamination risk may also increase with unsafe local practices such as intraoperative PMMA molding [9]. PMMA, which is more accessible in LMICs due to cost and workability, is more vulnerable to infections among cranioplasty materials [3]. Preformed PMMA implants show a reduced risk (0.66) as compared to hand-formed PMMA implants (10.98; relative risk = 1.05) [3]. Intraoperative implant preparation can also extend surgical time and introduce unpredictable variables such as mold fracture, as seen during dry sterilization in one reported case [9]. Materials that are biocompatible, sterilizable, and safe for human use must be available for medical applications; however, in LMIC surgical settings, these conditions are not verifiable due to a lack of quality control infrastructure. The safety of locally fabricated implants cannot be assured without material testing, post-processing validation, and quality control [8]. In many LMICs, there is no established framework for evaluating the safety and effectiveness of intraoperative molded prosthesis [8].

Sterilization compatibility should also be considered for 3D-printed implants. Different materials exhibit varying responses to autoclave and chemical sterilization, which can result in structural and biocompatibility implications. Autoclaving causes bends and cracks in stereolithography (SLA)-printed surgical guides, making them unreliable during operation [3]. FDM-printed models shrink 0.1%-3% after autoclaving, and PLA has a low melting point (180°C-220°C) [3]. Polyethylene terephthalate glycol (PET-G) and PMMA are also not suitable for autoclaving due to changes in their mechanical behavior after autoclaving. Therefore, they require alternative sterilization techniques such as ethylene oxide, hydrogen peroxide gas plasma, ultraviolet (UV), and gamma irradiation [3]. The printing process and repeated sterilization can affect the quality of materials by introducing defects related to residual stress, porosity, impurities, and lack of adherence [10]. The surface roughness of materials attracts harmful bacteria to implants and may promote biofilm formation. 3D-printed polymers with low surface roughness offer an advantage over conventional materials in reducing this risk [10]. Inertness, biocompatibility, eligibility for sterilization, greater strength, and long-term sustainability are some of the qualities of an ideal implant, but many conventional and reprocessed materials lack these qualities [4]. Sterile implants and instrumentation are not frequently available in LMICs, especially for neurosurgical procedures, which results in the adoption of improvised or reused materials [2]. Standard-sized imported implants and tools may not be accessible or affordable, but a customized implant on a per-print basis paves the way for personalized medicine [10]. Many hospitals lack access to imported disposables, but 3D-printed parts can bypass the complex supply chain and limitations of bulk manufacturing [10]. 3D printing can replace conventional manufacturing with its ability to construct designs with complex geometries, resulting in reduced dependence on expensive imported components [10]. Surgical instruments or tools can be created for specific patients going through a treatment. It may require a degree of personalization, which may not be possible with standard imported instrument sets [2].

Thirdly, personalized training for surgical procedures may also be done with 3D-printed models [1]. Currently, surgical trainees work on animals, cadavers, or plastic models. In low-income countries or non-university hospitals, obtaining corpses for surgical education is considered a major challenge [11]. Cadaver storage and treatment require a highly specialized center, and due to its associated cost and legal consequences, not all training centers have access to cadavers for surgical training [11]. Moreover, animals, cadavers, or plastic models lack realism and may not be suitable for practicing real-life surgery scenarios [2]. The variability of individual patients cannot be replicated using cadaveric specimens or general plastic models, as the human cerebrovascular system varies greatly [2]. Surgical education is transforming toward simulation-based learning [12]. Virtual reality can help in viewing aneurysms and surrounding anatomy, but it fails to provide real-life tactile sensations, and it cannot replicate the elasticity of tissue deformation [2]. Practicing on physical models can improve surgeons’ confidence and competence, and without these models, trainees would develop their skills directly on patients [11]. Multiple replicas from a single patient scan can be created using 3D-printed models, allowing as many repetitions of a surgical procedure as needed. Performing a high-risk procedure such as cervical fixation, pedicle screw insertion, and aneurysm clipping directly on a patient raises safety concerns [1]. Simulation-based training is now becoming more popular because the teaching faculty can concentrate on training during simulations and patient care during operations rather than conflicting the two [12]. Results from brain biopsy simulation studies also show that the absence of structured simulation practice or training greatly affects the learning of residents [12].

The systemic barriers affecting implant acquisition are highlighted in Table 1.

**Table 1:** Systemic barriers affecting neurosurgical implant access and utilization in low- and middle-income countries LMIC: low- and middle-income country, PMMA: polymethylmethacrylate, 3D: three-dimensional, DBS: deep brain stimulation, VNS: vagus nerve stimulation, SRS: stereotactic radiosurgery, MRgFUS: magnetic resonance-guided focused ultrasound, FNS: functional neurosurgery, CT: computed tomography, DICOM: Digital Imaging and Communications in Medicine, PLA: polylactic acid

Author and year of publication	Evidence type and Country of origin	Implant category	Standard material used	Reported constraint in LMIC literature	Clinical impact of constraint
Solomon et al. (2025) [7]	Systematic review, multiple LMICs	Cranial implants	PMMA (24.8%), titanium (8.32%)	High cost, limited resources, limited access to advanced implants, infrastructure limitations	Preferencing cost-effective methods, delay in cranioplasty
Asija et al. (2024) [13]	Observational study, LMIC	DBS, VNS, SRS, MRgFUS, rhizotomy	Not specified	Accessibility, affordability, lack of training	Reduced adoption of FNS techniques
Strelko et al. (2024) [14]	Pilot study, Philippines and Ukraine	Cranial implants	Not specified (focus on software design required for implants)	CT scan parameter variability, DICOM file array issues, technical usability challenges	Difficulty in creating accurate implants, limits immediate clinical scalability, cost-effectiveness
Ikwuegbuenyi et al. (2023) [15]	Narrative review, Tanzania	Spinal implant	Not specified	Affordability, lack of expertise, poor supply chain	Negative impact on patient care and best medical practice
Bečulić et al. (2023) [9]	Prospective observational study, Bosnia and Herzegovina	Cranial implant	PMMA	High cost of commercial implants, limited access to prefabrication services, resource limitation	Successful outcomes, cost effectiveness, reduction of complications
Ashraf et al. (2022) [6]	Retrospective case series, Pakistan	Cranial implants	PMMA, PLA, silicone mold	Resource limitations, limited access to prefabrication services	No major postoperative complications, excellent radiological and cosmetic outcomes, ultra-low-cost solution
Morales-Gómez et al. (2018) [16]	Case series, not specified	Cranial implants	PMMA	High cost of commercially available implant, limited accessibility of advanced implant solution	Excellent implant fit, improved aesthetic outcome, reduced cost

Leveraging 3D printing to tackle these challenges

3D printing has significantly improved accessibility and affordability compared to traditional cadaveric specimens and commercial simulation tools, thereby enhancing training and surgical planning opportunities [17]. Polymers such as PLA (20 USD per kg), PMMA (30 USD per kg), PET-G (40 USD per kg), and PEEK (>275 USD per kg) provide cost-effective biocompatible 3D-printed alternatives [3,17]. Furthermore, studies by Solomon et al. [7] and Ashraf et al. [6] demonstrated that the use of cost-effective 3D-printed PMMA implants for cranioplasty produced outcomes comparable to those achieved with other materials, such as titanium. Shilov et al. compared 3D printing using low-cost materials such as PLA and ABS with conventional manufacturing methods. They concluded that 3D printing is significantly more cost-effective while maintaining functional effectiveness [18]. The tensile strength of PEEK is 80 MPa, that of PMMA is 48-76 MPa, and that of PLA is around 47-52 MPa [3]. Elastic modulus (measure of stiffness) is also high in PMMA (3-5 GPa), PLA (3.4-5.7 GPa), and PEEK (3-4 GPa) [3]. The use of 3D printing technology has enhanced preoperative planning and improved understanding of patient-specific anatomy before surgery. Importantly, these tools are significantly more affordable than traditional cadaveric training and commercially produced simulators, making them particularly valuable in low- and middle-income settings [4]. Additionally, the use of low-cost and recycled materials further enhances the economic feasibility of 3D printing in neurosurgery. As a result, the integration of recycled filament production supports both cost reduction and resource efficiency, making 3D printing a more accessible and practical solution, particularly in low-resource healthcare settings [10].

Advantages of using recycled medical-grade plastics

High-purity medical-grade polymers, including polyethylene terephthalate (PET), polypropylene (PP), PLA, and high-density polyethylene (HDPE), are abundant in hospital waste streams such as intravenous (IV) fluid bottles, syringes, pharmaceutical packaging, and medical containers [19-21]. They all represent a great opportunity for sustainable fabrication of neurosurgical instruments and educational models via 3D printing. These polymers can be subjected to strict sterilization protocols, including autoclaving, chemical disinfection, and ultraviolet (UV) irradiation, effectively eliminating microbial contaminants while maintaining their intrinsic mechanical and chemical stability [20-23]. Post-sterilization, the materials are mechanically comminuted into uniform granules, thermally processed under controlled conditions, and molded into high-quality filaments that are compatible with additional manufacturing modalities such as FDM, SLA, and continuous liquid interface production (CLIP) [24].

The advantages of using recycled medical plastics extend beyond environmental considerations. Cost reduction is a significant benefit. Local recycling reduces dependence on imported commercial filaments and reduces supply chain restrictions in LMICs. Additionally, the approach promotes a circular economy by diverting potentially hazardous medical waste from landfills while simultaneously generating high-value, biocompatible products [19,21-23]. Local production allows rapid prototyping, patient-specific customization, and on-demand fabrication of surgical instruments, anatomical training models, and implants, addressing critical gaps in neurosurgical practice in resource-limited settings. Additionally, the flexibility of 3D printing enables multi-material designs with personalized mechanical properties, including optimized stiffness, elasticity, and fatigue resistance, which are essential for surgical instruments and scaffolds [22]. Collectively, the use of recycled medical-grade plastics integrates sustainability, affordability, and technical refinement, offering a sensible and environmentally responsible solution to support neurosurgical education, research, and clinical practice in LMICs [19,23,25].

Recyclable materials and methods

Each polymer exhibits elevated thermal stability, mechanical robustness, and biocompatibility, making them suitable candidates for conversion into 3D printing filament [5,20,22,23], for instance, polyethylene terephthalate (PET) derived from intravenous (IV) fluid bottles, PP sourced from syringes, PLA obtained from pharmaceutical packaging, and HDPE recovered from medical containers (Table 2) [25]. These can be systematically collected and segregated within hospital environments, thereby ensuring a sterile and controlled feedstock. The recycling protocol initiates with sterilization procedures to eradicate microbial contaminants, followed by mechanical comminution to generate granules of uniform dimensions, thereby facilitating precise thermal processing during filament extrusion [25,26].

Next, the polymer granules are heated to their specific melting points and formed into filaments with precise diameters, which can be used in 3D printing methods such as fused deposition modeling (FDM), direct writing, or stereolithography [23,24]. This process keeps the polymers’ strength and biocompatibility intact, allowing them to be reused multiple times while ensuring consistent print quality [25-27]. The process is very flexible, allowing different polymers to be mixed with additives to adjust properties such as strength, flexibility, and surface finish, which are important for making patient-specific neurosurgical implants, flexible surgical tools, and realistic anatomical models. In LMICs, this recycling method lowers costs and creates local manufacturing capabilities, thereby letting hospitals and universities make custom neurosurgical devices without relying on expensive imports. Moreover, it supports sustainable healthcare by turning potentially hazardous medical waste into useful clinical tools and educational models, hence combining environmental care with medical innovation.

**Table 2:** Global applications of medical-grade and recycled polymers in 3D-printed neurosurgical training and implant prototyping PLA: polylactic acid, FFF: fused filament fabrication, TPU: thermoplastic polyurethane, DD: direct drive, FDM: fused deposition modeling, PMMA: polymethylmethacrylate, CT: computed tomography, ABS: acrylonitrile butadiene styrene, PETG: polyethylene terephthalate glycol, CAD: computer-aided design, SLS: selective laser sintering, SLA: stereolithography apparatus, DLP: digital light processing

Author and year of publication	Study design/country	Polymer used	Material origin	3D printing technology	Neurosurgical evaluation	Mechanical/clinical validation	Cost/resource impact	Sustainability outcome
Sufianov et al. (2024) [28]	Cohort study, Russia	PLA	Recyclable	Open source (Autodesk Mesh mixer) FFF 3D printer	Used to replicate varying consistencies of spinal tissues, cervical spine	Low melting point and is advised to use low drill speed	Cheaper and easier to make	Recyclable supporting materials and minimal environmental footprint
Sidabutar et al. (2023) [29]	Pilot study, Indonesia	TPU	Synthetic and recyclable	DD modification onto Ender 3 Pro printer	Skull-based models, spinal models, and ventricular-based models	Reliable for patient-specific neuroanatomic constructs	Longer print time using low-cost flexible polymer	Not evaluated
Uhl et al. (2023) [11]	Case series, France	PLA	Recyclable	FDM	Cranioplasty in craniosynostosis	High accuracy and realism but can melt when drilled	Limited material availability and process is time-consuming	Not evaluated
Ashraf et al. (2022) [6]	Case series, Pakistan	PMMA (using food-grade silicon molds)	Recyclable	Open source (Autodesk Mesh mixer), Prusa i3 clone 3D printer (CT scan operated printer)	Cranioplasty	Excellent cosmetic appearance and patient satisfaction	40$ per implant, easier and considerably lower cost and accurate	Materials are obtained from local commercial shops
Czyżewski et al.(2022) [3]	Systematic review, Poland	PLA, ABS, PETG, PMMA	Recyclable	CAD software with FDM, SLS, SLA, and DLP	Cranioplasty	Satisfactory strength and low cost compared to other materials such as titanium	600$ and above for different materials, low material cost	Materials are obtained from local commercial shops
Scerrati et al. (2021) [30]	Observational study, Italy	PMMA (using silicon molds)	Synthetic and recyclable	VxElements, Meshmixer, Simply 3D	Cranioplasty	Affordable and consistency in production, poor realism	Low cost and easily shapeable	Silicon molds can be reused in case of infection or damage
Chaudhary et al. (2021) [31]	Observational study, Indonesia	TPU	Synthetic and recyclable	DD modification onto Ender 3 Pro printer	Skull-based models, spinal models, and ventricular-based models	Reliable for patient-specific neuroanatomic constructs	Longer print time using low-cost flexible polymer	Not evaluated

Cost comparison, safety, and technical considerations

Locally produced 3D-printed PMMA cranioplasty implants depict a more practical and cost-effective solution in low- and middle-income countries (LMICs) as compared to commercially prefabricated titanium or PEEK implants, which remain largely unaffordable [8,32].

Prefabricated implants may cost up to 10,000 USD. In contrast, locally produced PMMA implants can be manufactured at approximately 40 USD per patient, as reported by a Pakistani cohort, representing a cost reduction of over 90% [6,9]. The use of open-source software and low-cost 3D printers further helps in the production of training models and surgical simulation tools, providing similar economic benefits for education and skill development [6]. The surgeons trained on 3D models demonstrate better skills as per previous research [10].

Beyond the cost concerns, patient-specific PMMA implants demonstrate a much more favorable clinical, cosmetic, and radiological outcome when compared to freehand PMMA techniques [6]. In the reported series, most patients achieved excellent radiological alignment and high cosmetic satisfaction, with minimal complications [10]. However, technical considerations must be addressed carefully. Mechanical strength and structural integrity depend on accurate design and proper 3D printing; errors in modeling may lead to gaps or poor fitting, as observed in a single patient where the anterior implant did not align perfectly [7]. Biocompatibility of PMMA is generally good, and hence, the use of recycled or low-quality filament could introduce impurities. Additionally, heat resistance during sterilization is limited, requiring standard autoclaving at 121°C and 1 bar pressure to ensure safety, which does not confer a lot of benefit [6].

Despite these limitations, custom-designed implants can reduce intraoperative complications, improve cosmetic outcomes, and provide radiological precision far superior to conventional freehand PMMA molding [6]. The workflow using open-source software and in-office 3D printing is feasible for a single surgeon without formal IT training and is adaptable for public healthcare systems aiming to provide patient-specific solutions at minimal cost [6]. Overall, these methods demonstrate technological integration into neurosurgical practice.

Toward smart design: artificial intelligence ergonomics

The advent of artificial intelligence (AI) and its incorporation in medicine has drastically altered individuals’ approach to certain tasks. 3D printing was once thought to be pre-programmed, rigid, and static. By using neural networks, it has now become adaptive and stout. AI-driven models have successfully optimized temperature, layer height, flow, and surface texture [33]. A few examples include convolutional neural network (CNN) for imaging and visualization, recurrent neural network (RNN) for time sequential data, and artificial neural network (ANN) for functional optimization and prediction [34].

Patient-specific models demand precision and geometric intricacies. Machine learning (ML) algorithms improve the manufacturing process by limiting human design error. They can adjust the parameters at the micro-level; one such case is two-photon polymerization [35]. Furthermore, the real patient scenario mandates real-time decision-making. AI has transformed the printing process into an autonomous, data-driven process. Closed-loop deep learning (DL) models are being incorporated to achieve real-time optimization and predict material behavior. For instance, systems such as CAXTON use DL to fine-tune the parameters [35]. Although commercial acquisition is still in debate, AI models cut the cost by precision industrialization.

Implementation strategy and recommendations

The term plastic upscaling refers to the process of making high-end plastic products for regular usage from low-grade waste or undesirable materials. Different printing parameters influence the strength, brittleness, and dimensional accuracy of printed parts. It is extremely important to know the structural designs, properties of the materials, and machine parts for maximum optimization [7]. Plastic waste is segregated, and recycling includes primary, secondary, tertiary, and quaternary recycling [7]. For this purpose, we need to have strong interpersonal communication and exchange of ideas between engineers, manufacturers, and healthcare consumers.

**Figure 1:**
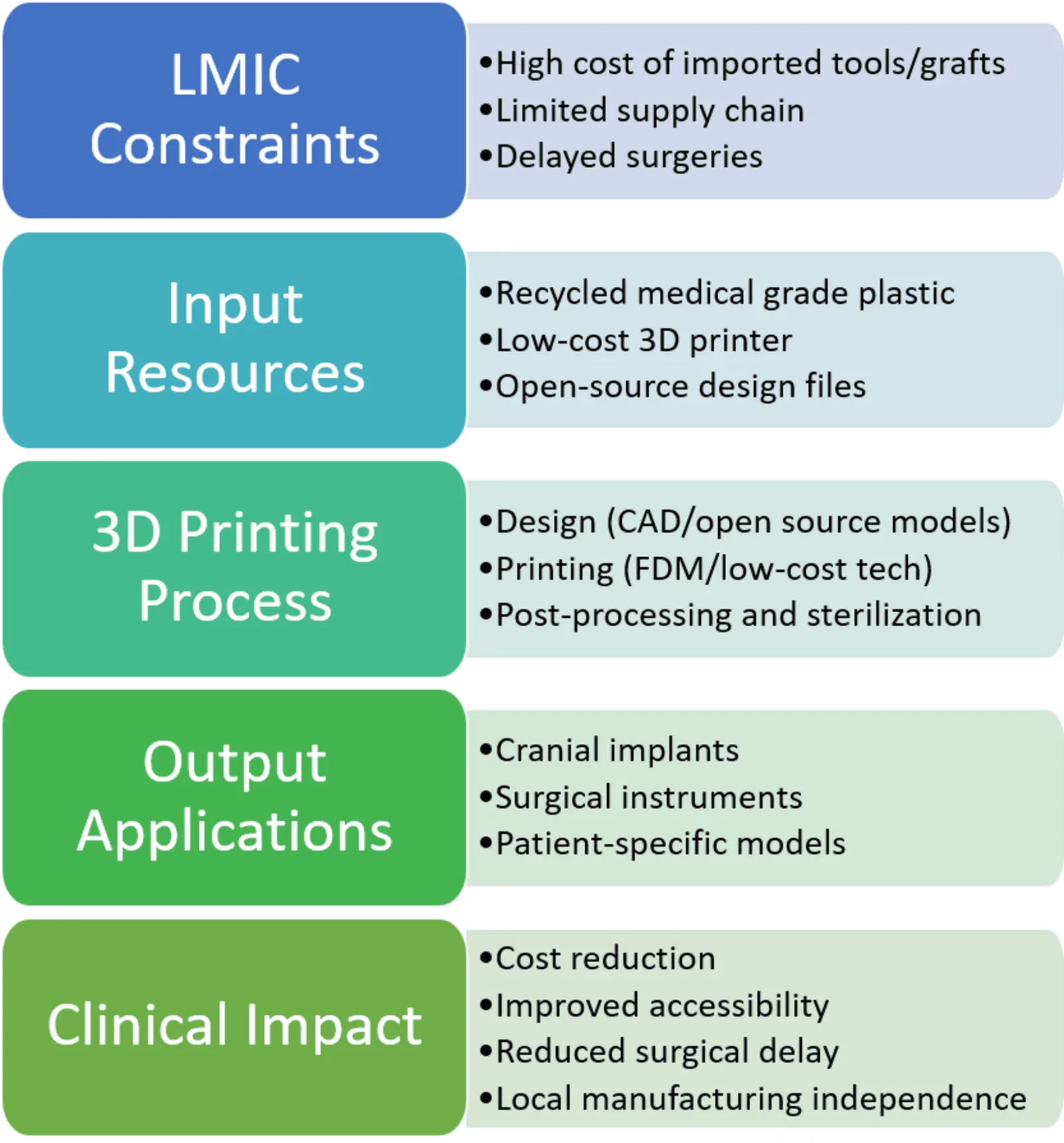
Author-proposed illustration of low-cost 3D printing pathway for neurosurgical solutions in LMICs LMICs: low- and middle-income countries, CAD: computer-aided design, FDM: fused deposition modeling Image created using Microsoft PowerPoint Image credit: Faiqa I. Khan

For any technology to be introduced in an LMIC public sector, the most crucial aspect is cost-effectiveness. This can be lowered significantly and effectively by using recycled plastic. The first step is to educate the healthcare administrative institutions about the segregation of plastic products in discrete echelons. Supplementary segregation should be introduced to locally recycle biodegradable plastic. The use of polymers is entirely based on the neurosurgeons’ opinion. It is economical to use this plastic instead of titanium or metal. The cost can be significantly cut down when large-scale plastics are recycled and used to make 3D products. Materials and filaments straight from synthetic manufacturers are also costly and have an expiry limit [36]. The applicability and affordability of end products have to be considered before ordering a 3D printer for an institute’s laboratory. To further ensure the reliability of the product, quality control standards ought to be redefined. Low-cost solutions enabled through 3D printing for LMICs are summarized in Figure 1.

Polymers such as PLA can be used to make prosthetics with outstanding similarity to the original material and can help neurosurgeons train. These would be low-cost and patient- and disease-specific, and would significantly improve the surgeon’s skills. These models will improve surgical planning and ensure simulation-based training. Moreover, the training of technicians and biomedical engineers has to be streamlined in order to keep the system working.

Limitations of the review

The present study is a narrative review, without formal bias assessment or Preferred Reporting Items for Systematic Reviews and Meta-Analyses reporting, which hinders its reproducibility. The literature on recycled biomaterials was scarce; hence, the intended conclusion was limited. Owing to the few studies, the compatibility, safety, and long-term additivity of recycled materials were not addressed properly, which limits their general applicability. Further, the cost analysis of recycling remains limited, which would impede policy makers to adapt this as a cost-effective alternative.

## Conclusions

3D printing has established its promising contribution in the neurosurgical field. Through the means of producing patient-specific implants, surgical tools, and anatomical models with high precision, low-cost biomaterials have already paved their way. The integration of recycled medical-grade plastics and polymer-based biomaterials offers a practical approach to overcoming financial barriers by boosting accessibility, proficiency, and local manufacturing capacity.

3D printing offers the potential for sustainable innovation in healthcare by utilizing locally available low-cost and recycled materials, aligning with cost-effective and environmentally conscious practices through the means of AI. Continued research, cross-disciplinary collaboration between clinicians, engineers, and material scientists, and the development of appropriate infrastructure will further enhance its role in advancing equitable neurosurgical care in LMICs.
